# Garlic-Derived Phytochemical Candidates Predicted to Disrupt SARS-CoV-2 RBD–ACE2 Binding and Inhibit Viral Entry

**DOI:** 10.3390/molecules30234616

**Published:** 2025-12-01

**Authors:** Martha Susana García-Delgado, Aldo Fernando Herrera-Rodulfo, Karen Y. Reyes-Melo, Ashly Mohan, Fernando Góngora-Rivera, Jesús Andrés Pedroza-Flores, Alma D. Paz-González, Gildardo Rivera, María del Rayo Camacho-Corona, Mauricio Carrillo-Tripp

**Affiliations:** 1 Biomolecular Diversity Laboratory, Centro de Investigación y de Estudios Avanzados del Instituto Politécnico Nacional Unidad Monterrey, Vía del Conocimiento 201, PIIT, Apodaca 66600, Nuevo León, Mexico; 2 Facultad de Ciencias Químicas, Universidad Autónoma de Nuevo León, Avenida Universidad s/n, Ciudad Universitaria, San Nicolás de los Garza 66455, Nuevo León, Mexico; 3 Facultad de Medicina, Universidad Autónoma de Nuevo León, Av. Dr. José Eleuterio González 235, Mitras Centro, Monterrey 64460, Nuevo León, Mexico; 4 Facultad de Agronomía, Universidad Autónoma de Nuevo León, Francisco Villa S/N, Col. Exhacienda El Canadá, General Escobedo 66050, Nuevo León, Mexico; 5 Laboratorio de Biotecnología Farmacéutica, Centro de Biotecnología Genómica, Instituto Politécnico Nacional, Reynosa 88710, Tamaulipas, Mexico

**Keywords:** *Allium sativum*, pharmacokinetic profiling, drug-likeness, bioactive metabolite identification, computational drug discovery

## Abstract

The emergence of SARS-CoV-2 and its rapid global spread underscores the urgent need for novel therapeutic strategies. This study investigates the antiviral potential of *Allium sativum* (garlic) extracts against SARS-CoV-2, focusing on disruption of the spike protein’s receptor-binding domain (RBD) interaction with angiotensin-converting enzyme 2 (ACE2), a critical step in viral entry. Two garlic cultivars (Tigre and Fermín) were processed via oven-drying or freeze-drying, followed by maceration with CH_2_Cl_2_/MeOH (1:1) and fractionation with liquid–liquid partition. ELISA immunoassays revealed that freeze-dried Tigre (TL) extracts had the highest inhibitory activity (42.16% at 0.1 µg/mL), with its aqueous fraction achieving 57.26% inhibition at 0.01 µg/mL. Chemical profiling via GC-MS found sulfur and other types of compounds. Molecular docking identified three garlic TL-derived aqueous fraction compounds with strong binding affinities (ΔG = −7.5 to −6.9 kcal/mol) to the RBD-ACE2 interface. Furthermore, ADME in silico analysis highlighted one of them (L17) as the main candidate, having high gastrointestinal absorption, blood–brain barrier permeability, and compliance with drug-likeness criteria. These findings underscore garlic-derived compounds as promising inhibitors of SARS-CoV-2 entry, calling for further preclinical validation. The study integrates experimental and computational approaches to advance natural product-based antiviral discovery, emphasizing the need for standardized formulations to address therapeutic variability across viral variants.

## 1. Introduction

The coronavirus disease of 2019 (COVID-19) was declared a pandemic by the World Health Organization (WHO) on 11 March 2020. The causative agent is the severe acute respiratory syndrome coronavirus 2 (SARS-CoV-2). As of 25 February 2024, over 700 million confirmed cases and more than 7 million deaths have been reported globally [1]. Among the seven known human coronaviruses (HCoVs), SARS-CoV (identified in China in 2002) and the Middle East respiratory syndrome coronavirus (MERS-CoV, identified in Saudi Arabia in 2012) caused prior epidemics. Insights from these outbreaks facilitated the rapid development of containment strategies, vaccines, and therapies for COVID-19. SARS-CoV-2 is an enveloped +ssRNA betacoronavirus with a ~30 kb genome and a spherical virion of 80–120 nm. Its defining feature is the trimeric spike (S) glycoprotein, where the S1 subunit binds ACE2 receptors, and S2 drives membrane fusion. This makes the RBD, particularly its receptor-binding motif, a prime therapeutic target. Additional drug strategies focus on inhibiting viral proteases (PLpro, Mpro) and the RNA-dependent RNA polymerase needed for replication [2,3].

To date, COVID-19 treatments are classified into five primary medication categories: antivirals, immune modulators, anticoagulants, monoclonal antibodies (mAbs) targeting SARS-CoV-2, and renal replacement therapies. Among these, only antivirals and SARS-CoV-2-specific mAbs directly target the virus, whereas other groups mitigate the disease complications. As of 2024, the United States Food and Drug Administration (FDA) has approved two antivirals for COVID-19 treatment: Paxlovid (a combination of nirmatrelvir and ritonavir) and Veklury (remdesivir). Lagevrio (molnupiravir) remains authorized solely for emergency use [4]. Paxlovid inhibits the SARS-CoV-2 main protease (Mpro), with nirmatrelvir directly blocking viral protease activity to prevent replication. Ritonavir, a cytochrome CYP3A inhibitor, prolongs nirmatrelvir’s efficacy by reducing its metabolic degradation [5].

Remdesivir, an adenosine analog prodrug, is metabolized intracellularly to its monophosphoramidate form and subsequently converted into an active adenosine triphosphate analog. It impedes viral replication by binding to the RNA-dependent RNA polymerase (RdRp), inducing premature termination of RNA transcription [6]. Molnupiravir, another prodrug, undergoes rapid conversion in plasma to β-D-N4-hydroxycytidine (NHC). Host kinases phosphorylate NHC into its triphosphate form (NHC-TP), which is incorporated into the viral genome by RNA polymerase. This incorporation introduces lethal mutations, thereby suppressing viral replication [5].

Regarding monoclonal antibodies (mAbs), none have received FDA approval to date, though several remain under investigation in clinical trials. While these antibodies have demonstrated therapeutic potential against SARS-CoV-2 infection, their efficacy varies markedly among viral variants and subvariants. This variability has delayed regulatory approval and underscores concerns about their universal effectiveness against all SARS-CoV-2 strains [4]. Notably, most molecules tested in SARS-CoV-2 clinical trials are repurposed from prior antiviral or disease research, with no novel compounds specifically developed for this virus.

The challenges posed by SARS-CoV-2’s genetic diversity (over 75 documented variants; [7]), the absence of new antiviral agents, and the anticipated emergence of novel human coronaviruses (HCoVs) necessitate continued exploration of alternative therapeutics. Natural products (NPs) represent a critical strategy in this pursuit, given their historical significance in drug discovery, particularly for infectious diseases. NPs exhibit unique advantages, including structural diversity and molecular complexity. Furthermore, their origins in traditional medicinal sources provide preliminary insights into efficacy and safety profiles, though rigorous pharmacological validation remains essential [8].

During the COVID-19 pandemic, populations globally turned to traditional medicine to mitigate the disease. *A. sativum* L. (Amaryllidaceae), commonly known as garlic, emerged as one of the most widely utilized remedies, often consumed alone or in combination with botanicals such as ginger, turmeric, chamomile, cinnamon, lime, lemon, black pepper, artemisia, neem leaf, orange, onion, and cardamom [9,10,11,12,13,14,15]. Emerging evidence suggests a correlation between dietary garlic intake and reduced risk of severe COVID-19 outcomes [16]. However, the lack of standardization in the preparation methods and dosages raises concerns, as excessive consumption may pose health risks. Current guidelines recommend a maximum daily intake of 4 g raw garlic for adults or 50 mg/kg body weight [17,18,19]. Consequently, identifying and quantifying garlic’s bioactive compounds is critical to developing standardized extracts with defined antiviral activity. Such formulations could serve as adjuvants to reduce the viral load in COVID-19 patients or enhance the therapeutic efficacy.

Garlic is a bulbous herbaceous plant widely utilized both as a culinary spice and a medicinal agent. Recognized as one of the oldest herbs employed since ancient times in traditional medicine, it has historically been used to treat parasitic infections, rheumatism, dermatological conditions (e.g., scabies, warts), animal or insect bites, and respiratory ailments such as cough, asthma, bronchitis, hoarseness, and influenza [20,21]. Garlic is classified as a functional food due to its broad pharmacological properties, including antibacterial, antifungal, antiprotozoal, antiviral, antioxidant, anti-inflammatory, anticancer, anti-Alzheimer’s, and antihypertensive activities [22]. Furthermore, garlic exhibits therapeutic activity against metabolic disorders such as obesity, diabetes mellitus, and dyslipidemia, which are comorbidities associated with severe COVID-19 outcomes [2,20,23,24,25,26,27].

Being among the most rigorously studied medicinal plants, garlic has garnered significant interest in its potential as a preventive agent and adjunct therapy against viral infections, including SARS-CoV-2. Key pharmacological properties relevant to COVID-19 management include immunomodulatory, antioxidant, anti-inflammatory, and antihypertensive effects. It has been proposed that this stems from the bioactive diversity of its extracts and unique organosulfur compounds. Table 1 shows some previously reported potential anti-SARS-CoV-2 compounds and their target, either the viral protease MPro or the cell’s ACE2 receptor.

In the present work, we aimed to investigate *A. sativum* whole extracts and their fractions as potential sources of antiviral molecules for developing novel therapeutics against human coronaviruses, particularly SARS-CoV-2. We focused our study on the receptor binding domain of the viral Spike, given its implications in the first steps of the infection process. To elucidate the antiviral mechanisms, we integrated experimental and computational methodologies, enabling detailed insights into modes of action. This dual approach helped the precise identification of compounds with therapeutic potential, providing foundational data to guide their development as agents against COVID-19.

## 2. Results

### 2.1. Garlic Bioactive Compounds

Two garlic varieties were analyzed under distinct drying conditions. The total extracts and fractions were evaluated via ELISA immunoassays. Although none exhibited ≥50% inhibition, the inhibitory concentration (IC) was determined through data interpolation, and the maximum inhibition values were recorded to assess the relative efficacy (Table 2). Among the four extracts—Fermín freeze-dried (FL), Fermín oven-dried (FE), Tigre freeze-dried (TL), and Tigre oven-dried (TE)—the TL extract showed the highest potency, achieving 42.16% of maximum inhibition (with an IC_35_ of 0.1 μg/mL).

TL’s inhibitory activity at low concentrations underscored its potential as a candidate for further investigation. The TL extract was subjected to sequential liquid–liquid partitioning using solvents of varying polarities such as hexane, chloroform, and ethyl acetate, yielding four fractions. These fractions were serially diluted to concentrations of 100, 10, 1, 0.1, 0.01, and 0 µg/mL for subsequent immunoassays analysis.

Table 3 summarizes the inhibitory potential of the four fractions (hexane, chloroform, AcOEt, and aqueous) derived from the TL extract. Notably, the aqueous fraction achieved the highest inhibition of 57.26% (IC_40_ of 0.01 μg/mL), indicating its potent inhibitory capacity at low concentrations. This result positions the aqueous fraction as the most promising candidate among those evaluated, with its constituents harboring bioactive compounds capable of effectively inhibiting the quaternary RBD interface of SARS-CoV-2. As a comparison with a known control reference, similar immunoassays were carried out for methylene blue, which has been reported to inhibit the SARS-CoV-2 Spike-ACE2 protein–protein interaction [31], obtaining a maximum inhibition of 63.90% at a concentration of 1000 µg/mL.

### 2.2. GC-MS Analysis of TL Bulbs Aqueous Fraction

The GC-MS analysis results of the TL-derived aqueous fraction are shown in Table 4, with general percentages of the following compounds: sugar (2.48%), sulfur compounds (14.48%), organic acids (9.54%), esters (11.83%), aminoacids (6.47%), and others (55.20%).

### 2.3. Molecular Docking

The RBD of the SARS-CoV-2 S protein serves as a pivotal structural component for viral entry, mediating host cell infection through its interaction with ACE2. Disrupting this interaction represents a promising therapeutic strategy to block viral pathogenesis. In this study, ELISA immunoassays combined with GC-MS analyses identified bioactive compounds in *A. sativum* from the TL-derived aqueous fraction with potential inhibitory activity. A total of 55 GC-MS identified phytochemicals (Table 4) were analyzed by molecular docking to evaluate their steric and electronic complementarity at the quaternary RBD–ACE2 binding interface.

Hence, to define the docking search space, we focused on the RBD region of the SARS-CoV-2 spike protein that directly contacts ACE2, i.e., the same interface interrogated by the ELISA RBD–ACE2 binding inhibition immunoassay. We evaluated the viability of this interface using pocket- and hotspot-identification approaches conceptually related to geometric pocket mapping (CASTp), ligand-binding hotspot clustering (FTMap), and druggability scoring of shallow concave surfaces (PockDrug). Specifically, the PockDrug analysis [32] identified a shallow but contiguous depression at the ACE2-contacting surface of the RBD, enriched in both hydrophobic and hydrogen bond-capable residues and predicted to contain ligandable hotspots (pocket P1) with Volume Hull = 540.65 Å^3^ and 12 pocket residues. Other features (P2–P4) scored lower, with P2 considered a decoy. Guided by this result and by the ELISA’s interface specificity, we centered the docking grid on the ACE2-contacting region at (X, Y, Z) = (−47.398, −20.053, 17.466) with dimensions 24 × 44 × 32 Å (grid spacing 0.375 Å), fully encompassing P1 and neighboring residues within ~10 Å. The identified cavity overlaps with the functional ACE2 contact patch, supporting its biological relevance. We therefore restricted our docking to this rationally predicted interface pocket rather than performing unrestricted (‘blind’) docking over the entire S1 spike protein surface.

**Figure 1:**
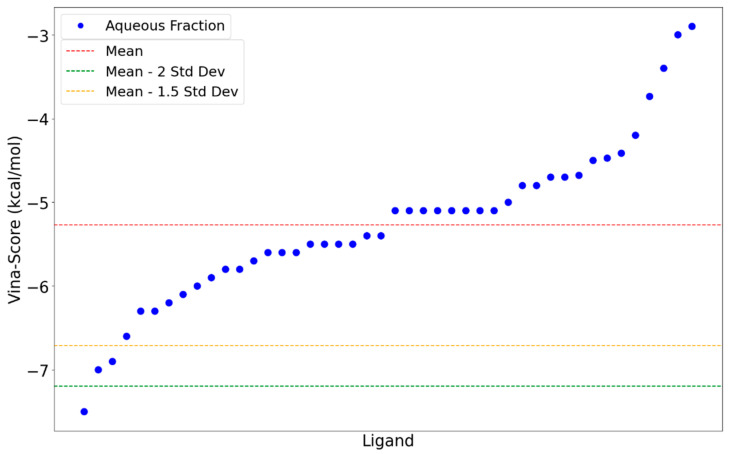
AutoDock Vina scores of TL-derived aqueous fraction compounds in complex with RBD [kcal/mol]. Statistical metrics are indicated by dashed horizontal lines: average score (red), mean minus one and a half standard deviation (orange), and the mean minus two standard deviations (green).

Using AutoDock Vina software v1.2.7, compounds were docked into the predefined RBD quaternary interface, and binding affinities were ranked based on an energy score [kcal/mol]. This computational approach aimed to identify high-affinity compounds capable of competitively inhibiting the RBD–ACE2 interaction. Data analysis was performed to prioritize compounds based on binding affinity scores. AutoDock Vina-derived affinity scores incorporate key physicochemical parameters—Van der Waals interactions, electrostatic forces, hydrogen bonding, solvation effects, and torsional entropy—expressed numerically. These values quantify RBD-compound binding energetics, where more negative scores indicate significantly stronger compound affinity for the RBD relative to its mean binding affinity across all evaluated ligands. Conversely, less negative scores denote weaker-than-average interaction strength. This comparison enables identification of compounds with preferential binding specificity for the RBD-ACE2 interface, independent of absolute affinity magnitudes (Figure 1).

**Figure 2:**
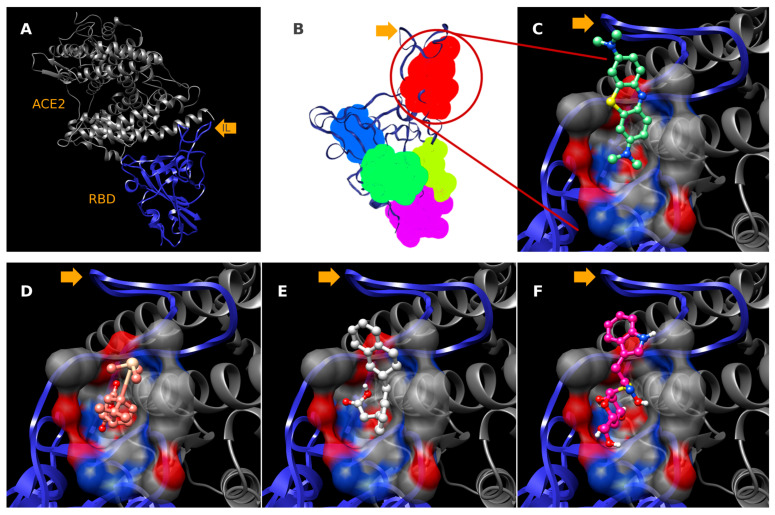
Binding-site rationale and docking poses at the SARS-CoV-2 RBD–ACE2 interface. (**A**) Overall RBD–ACE2 complex (RBD in blue, ACE2 in grey; cartoon representation). The orange arrow marks the interface loop (IL) used as a positional reference throughout. (**B**) Druggability mapping of the RBD surface using PockDrug analysis; the most likely ligandable pocket (P1, red) lies on the ACE2-contacting face; additional lower-scoring surface features are shown in blue/green/magenta. (**C**–**F**) Best-scoring docking poses. P1 is rendered as a semi-transparent molecular surface color-coded by atom type (carbon grey, oxygen red, nitrogen blue). (**C**) Methylene blue (experimental control), (**D**) Candidate L17, (**E**) Candidate L20 and, (**F**) Candidate L36. All docked ligands occupy the same interface-proximal depression identified as P1 in panel (**B**).

Three garlic-derived compounds with binding scores below the threshold of −2.0 standard deviations from the mean were identified as the most probable mediators of the observed ELISA inhibitory activity via disruption of the RBD–ACE2 interaction: compound L36 (−7.5 kcal/mol), L20 (−7.0 kcal/mol), and L17 (−6.9 kcal/mol). These compounds showed the most negative binding Vina scores within the analyzed dataset. Furthermore, we verified that the poses of these three top-ranked candidates lie within the hotspot-enriched depression identified by our druggability assessment. This supports the interpretation that these phytochemicals are not being artificially forced into an implausible flat surface but, instead, occupy a region with predicted small-molecule ligand ability at the RBD–ACE2 interface (Figure 2), potentially hindering the formation of the infection-initiating complex as a putative molecular mechanism that explains the ELISA immunoassays results.

## 3. Discussion

Our analyses revealed that garlic extracts and fractions are primarily composed of organosulfur compounds (OSCs), fatty acid esters, aldehydes, and ketones. OSCs exhibit broad-spectrum antiviral activity against pathogens such as influenza, rhinovirus, and adenovirus, among others [22]. Recent studies highlight their potential anti-SARS-CoV-2 activity, with multiple mechanisms of action elucidated. The predominant mechanism involves inhibition of the major protease (Mpro), a key enzyme in SARS-CoV-2 replication and viral packaging [30]. Structural studies further demonstrate that specific OSCs directly bind Mpro, disrupting their function [28]. These findings underscore OSCs’ multifaceted role in targeting critical viral components.

For instance, γ-L-glutamyl-S-allylcysteine—reported in a molecular docking study by Parashar et al. [30]—exhibits high estimated affinity (18.7 μM–1.86 mM) for SARS-CoV-2 Mpro. Similarly, allicin, investigated in an in silico study by Shekh et al. [29], acts as a potent Mpro inhibitor via dual S-thioallylation of the solvent-exposed Cys-145 and Cys-85/Cys-156 residues. In contrast, no literature evidence supports anti-SARS-CoV-2 activity for fatty acids or fatty acid esters identified in this study’s extracts and fractions. A prior molecular docking analysis of volatile compounds from *Chorisia* tree species [33], assessed interactions with SARS-CoV-2 structural proteins, but none overlapped with compounds from *A. sativum*. While omega-3 fatty acids have been implicated in immune modulation during SARS-CoV-2 infections [34], these were absent in the analyzed extracts. Additionally, no anti-SARS-CoV-2 activity has been reported for amino acids, ketones, aldehydes, or other compound classes detected in this study’s *A. sativum* fractions. However, our GC–MS analysis of the aqueous extract of *A. sativum* revealed compounds with other reported biological activities, notably antioxidant and anti-inflammatory effects (Table 5).

### 3.1. Evaluation of Garlic Anti-SARS-CoV-2-RBD Therapeutic Potential

Nonetheless, this study’s principal contribution lies in having identified three *A. sativum* TL-derived aqueous fraction compounds (L36, L20, L17) with potential inhibitory activity against SARS-CoV-2. Mechanistically, these ligands likely disrupt the quaternary RBD–ACE2 interface, a critical interaction for viral entry. Integrated methodologies—ELISA immunoassays, GC-MS analyses, and molecular docking—revealed strong binding affinities for these compounds, as reflected by favorable free energy scores (ΔG = −7.5 to −6.9 kcal/mol). These findings position these garlic phytochemicals as promising compounds for developing antiviral therapeutics targeting the initial stages of SARS-CoV-2 infection.

### 3.2. Pharmacokinetic Profiles and Toxicity In Silico

The physicochemical and pharmacokinetic properties of the three candidate compounds were systematically evaluated to assess their potential as drug-like compounds. The comparative data are summarized in Table 6. L36 displayed high polarity, as indicated by its elevated number of hydrogen bond acceptors (7) and donors (6). This polarity was reflected in a low consensus Log P value (0.18) and a favorable solubility profile (Ali class: soluble). However, these properties translated into poor gastrointestinal (GI) absorption and lack of blood–brain barrier (BBB) permeability. Moreover, L36 violated multiple drug-likeness rules (Lipinski, Ghose, Veber, Egan, and Muegge) and showed a low bioavailability score (0.55). The predicted inhibition of CYP1A2 suggested possible drug–drug interactions, and the compound was further limited by its relatively high synthetic complexity. Overall, L36 appears unsuitable as an oral drug candidate.

L20 demonstrated a favorable profile. With only one hydrogen bond donor and two acceptors, it exhibited higher lipophilicity (consensus Log P: 4.14), leading to good membrane permeability, although at the cost of reduced solubility (Ali class: moderately soluble). Importantly, L20 showed high GI absorption and BBB permeability, with a superior bioavailability score of 0.85. While it was predicted to inhibit CYP2C9 and CYP2D6, which could lead to metabolic interactions, it had no PAINS or Brenk alerts and showed minimal drug-likeness violations (only one Muegge violation).

Furthermore, it was synthetically accessible (score: 2.77), supporting its potential for development. Although compound L20 showed excellent gastrointestinal absorption and the highest predicted bioavailability, its low aqueous solubility and inhibition of several CYP isoforms raise concerns about metabolic stability, potential drug–drug interactions, and overall druggability.

L17 presented a balanced profile, with moderate lipophilicity (consensus Log P: 2.49) and good solubility (Ali class: soluble); it showed high GI absorption, BBB permeability, and, notably, no inhibition of cytochrome P450 isoforms, indicating a lower likelihood of drug–drug interactions. L17 complied fully with all major drug-likeness rules and was free of PAINS alerts. However, its bioavailability score was moderate (0.55), and it showed a high synthetic accessibility score (4.91), suggesting challenges in practical synthesis. Despite these limitations, the combination of favorable attributes, acceptable lipophilicity, and overall safety profile designate L17 as the most favorable candidate among the three, with strong potential for further optimization and development.

In summary, the comparative analysis highlights L17 as the most promising candidate, owing to its favorable absorption, solubility, absence of CYP inhibition, and full compliance with drug-likeness rules. L20, while synthetically more accessible and showing high bioavailability, is limited by poor solubility and CYP-related liabilities, making it a less suitable candidate. Conversely, L36 demonstrated significant limitations that reduced its potential for further development.

**Figure 3:**
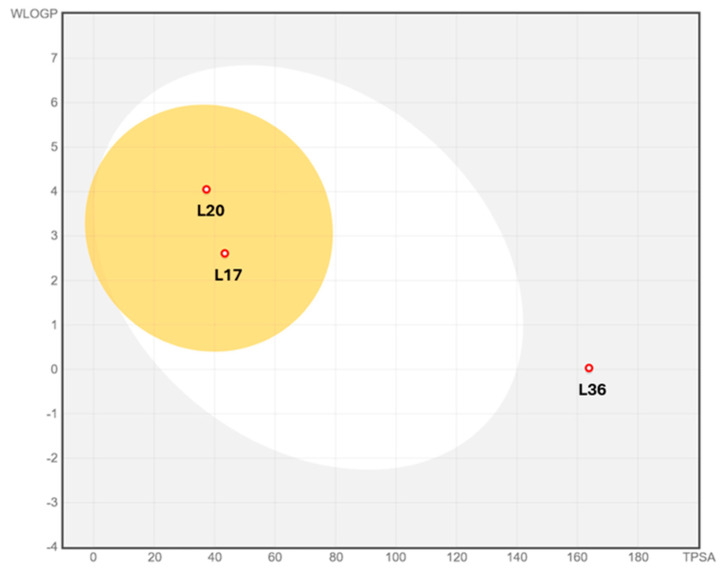
BOILED-Egg model of compounds (L36, L20, L17).

The pharmacokinetic properties of the three compounds were further assessed using the BOILED-Egg model (Figure 3), which predicts gastrointestinal absorption (GI) and BBB penetration based on WLOGP and TPSA values. L36 positioned outside both the white and yellow regions, exhibited a high TPSA (~160 Å^2^) and low lipophilicity, indicating poor GI absorption and negligible BBB permeability, consistent with its unfavorable ADMET profile. In contrast, L20, located within the yellow region, displayed optimal polarity (TPSA ~ 40 Å^2^) and lipophilicity (WLOGP ~ 4), suggesting both high GI absorption and strong BBB penetration. This observation supports its superior bioavailability and drug-likeness properties identified earlier. L17, with moderate lipophilicity (WLOGP ~ 2.5) and TPSA (~45 Å^2^), was also positioned within the yellow region, indicative of good oral absorption and potential, though less certain, BBB penetration. These findings reinforce the comparative analysis, where L20 appeared as the most promising candidate for oral administration and CNS activity, L17 stood for a balanced scaffold with favorable absorption and limited metabolic liabilities, and L36 was believed unsuitable due to its excessive polarity and poor permeability.

Toxicity predictions revealed distinct safety profiles for the three compounds (Table 7). Compound L36 showed the highest toxicological concern, with active predictions for nephrotoxicity (0.57), respiratory toxicity (0.74), and mutagenicity (0.50). The presence of mutagenic potential in L36 is of particular concern, as mutagenicity is a high-risk endpoint directly associated with genotoxic effects and long-term carcinogenic implications. Although L20 avoided mutagenicity, the simultaneous presence of neurotoxicity, nephrotoxicity, and ecotoxicity concerns could limit its suitability for further use. In contrast compound L17 showed fewer active toxic endpoints compared to L36 and L20, being positive for respiratory toxicity (0.67), BBB penetration (0.89), and ecotoxicity (0.59). Importantly, L17 was inactive for both nephrotoxicity and mutagenicity, two endpoints regarded as critical determinants in preclinical safety evaluation. Cytotoxicity was predicted as inactive with a high probability (0.79), indicating strong confidence that L17 is unlikely to be cytotoxic. Additionally, although L17 is predicted to cross the blood–brain barrier (0.89), its neurotoxicity is classified as inactive (0.70), suggesting that despite CNS exposure, it is predicted to be unlikely to cause harmful effects on the nervous system. Overall, these findings suggest that L17 has the most favorable toxicological profile among the three compounds, indicating a higher safety margin and greater suitability for further pharmacological development.

## 4. Materials and Methods

### 4.1. Garlic Cultivation and Extraction

*A. sativum* L. cultivation, varieties Tigre (T) and Don Fermín (F, speckled type), was established in the town of La Ascensión, Aramberri, Nuevo León, Mexico, at 24°21′90″ N, 99°56′24″ W and an altitude of 1960 m above sea level. Planting took place in October 2021, prior to the start of the cold season. Sowing was carried out manually, using a seeding density of 1200 kg of seed per hectare. The seed had previously received a chemical treatment, consisting of moistening it with a water-based mixture containing cypermethrin and carbofuran insecticides, as well as tetracycline and copper tetracycline to prevent attacks from insects, fungi, and soil bacteria. An auxin-type plant hormone was also applied to ensure proper crop establishment. The seed was distributed in the field in wide rows spaced 1.20 m apart, with two rows planted 0.05 m apart, resulting in a plant density of 330,000 plants per hectare. Soil moisture was supplied (irrigation) during crop development through a pressurized drip tape system, drawing water from a depth of 100 m. Mineral nutrition management consisted of applying 120 kg of nitrogen per hectare to the soil, using 130 kg of urea (46% nitrogen) and 292 kg of ammonium sulfate (20.5% nitrogen). Additionally, 60 kg of phosphorus was supplied using 190 kg of phosphoric acid (31.6% phosphorus) as the source. During the growth and development of the crop, pyrethroid insecticides are used to manage insect pests; for the control of fungal and bacterial pathogens, foliar applications of water with tetracycline and water with tetracycline-Cu, respectively, are used. Bulb harvesting takes place when 5 to 10% of the plants have exposed inflorescences. The plants, including their roots, are manually extracted from the soil using an agricultural implement called a “wide-winged plow.” The plants are then piled on the soil surface to release moisture until they reach approximately 60% hydration. The foliage and roots were then removed, leaving only the bulbs (garlic heads), the harvestable structures. These were placed in slotted plastic boxes to allow for aeration, reaching approximately 30% hydration. They were then sorted by total volume and packaged [64].

From the harvested bulbs, 910 g (T) and 890 g (F) of raw garlic were obtained. After peeling, the cloves yielded 675 g (T) and 750 g (F) of processed bulbs. These were sliced and subjected to two drying methods: oven drying (Quincy-Lab Inc Berlin, Germani), (E) at 35 °C for 120 h and freeze-drying (Labconco Corporation 8811, Prospect Ave. Kansas City, MO, USA) (L) at −52 °C under 0.63 mBar. The sliced T bulbs were divided into two 337.5 g batches, and the F bulbs into two 375 g batches. Oven drying produced 110.2 g (TE) and 124.1 g (FE) of dried material, while freeze-drying yielded 113.85 g (TL) and 135.9 g (FL). All batches were ground using a blade mill. Each dried sample underwent five sequential extractions with 400 mL of a 1:1 (*v*/*v*) dichloromethane (CH_2_Cl_2_)/methanol (MeOH) mixture. Maceration was performed for over 48 h at room temperature with intermittent stirring. The extracts were vacuum-filtered, and the filtrates were distilled under reduced pressure. Residual solvents were removed using a nitrogen stream, yielding the following dried extracts: TE (4.2363 g; 3.84% yield), FE (5.8417 g; 4.71%), TL (5.93 g; 5.21%), and FL (5.04 g; 3.71%). All extracts were stored at −20 °C for subsequent analysis.

### 4.2. Fractioning of Crude Extracts

Three grams of the most active extract were fractionated via sequential liquid–liquid partitioning using hexane (Hex), chloroform (CHCl_3_), and ethyl acetate (AcOEt). Each fraction was treated with anhydrous sodium sulfate, filtered to remove the desiccant, and concentrated under reduced pressure. The residual solvents were evaporated under a nitrogen stream, yielding the following dried fractions: 1.2 g (hexane), 0.03 g (CHCl_3_), and 0.012 g (AcOEt). While the aqueous fraction was lyophilized yielding 1.395 g (aqueous). The immunological activity of the fractions was assessed by ELISA. The active fractions (aqueous) were analyzed using Gas Chromatography–Mass Spectrometry (GC-MS).

### 4.3. ELISA Immunological Assays

High-binding-capacity polystyrene 96-well plates (flat bottom, 350 µL volume) were utilized for the assay. Antigen immobilization was performed by diluting SARS-CoV-2 Spike S1 protein (Sino Biological, Cat. #40591-V08H3, Houston, TX, USA) to 100 µg/mL in 0.2 M carbonate/bicarbonate buffer (pH 9.4). Then, 100 µL of this solution was added to each well, followed by an 18 h incubation at 4 °C under agitation. After coating, the plate underwent four washes (300 µL per well) with Wash Buffer (1xTBS, 0.1% Tween 20, St. Louis, MO, USA), each lasting 5 min at room temperature under agitation. Blocking was performed with 300 µL/well of 1% bovine serum albumin (BSA) in 1xTBS, and the mixture was incubated for 1 h at room temperature with shaking. After blocking, the plate was treated with 100 µL/well of the four garlic extracts (FE: Fermín oven-dried; FL: Fermín freeze-dried; TE: Tigre oven-dried; TL: Tigre freeze-dried). These extracts were applied at concentrations of 0.01, 0.1, 1, 10, 100, and 1000 µg/mL, in triplicate per concentration. The plate was then incubated for 2 h at room temperature with continuous agitation. Following incubation, the plate was washed as previously described. Additionally, the fractions: hexane, chloroform, ethyl acetate (AcOEt), and aqueous were evaluated using the same procedure. These fractions were tested at concentrations of 0.01, 0.1, 1, 10, and 100 µg/mL.

A total of 100 µL per well of the diluted SARS-CoV-2 Spike antibody (Sino Biological, Cat. #40150-D001-H) was added to the plate and incubated for 1 h at room temperature with gentle agitation. After six washes with Wash Buffer, 100 µL per well of 3′,5,5′-tetramethylbenzidine substrate solution (1-Step^TM^ Turbo TMB-ELISA, Cat. #34022, Thermo Scientific, Waltham, MA, USA) was added and incubated for 30 min at room temperature in the dark. The reaction was stopped by adding 100 µL per well of hydrochloric acid (HCl), and the absorbance was immediately measured at 450 nm using a spectrophotometer. To produce a standard curve, the antibody was diluted to 6.25, 12.5, 25, 50, 75, and 100 ng/mL in buffer (1xTBS, 0.05% Tween 20, 0.5% BSA). A total volume of 305 µL was prepared to accommodate triplicate measurements for each standard concentration, and the absorbance was measured at 450 nm using a spectrophotometer (Biotek Instruments ELx800, Santa Clara, CA, USA).

Raw absorbance A450 values for each extract/fraction concentration were acquired in triplicate and averaged (Ā450_c_). Prior to normalization, readings were blank-corrected using reagent blanks run on the same plate. The resulting means were then normalized to the maximum binding signal of the antibody standard curve (top standard), which was defined as 100% RBD–antibody binding (Ā450_max-std_). The percent binding for each condition was calculated as (Ā450_c_/Ā450_max-std_) × 100. The percent inhibition reported in the Results section was computed as

% inhibition = 100 × [1 − (Ā450_c_/Ā450_max-std_)].

For each concentration, the values are presented as the mean ± standard error (SE) from 3 independent wells and 5 independent experiments performed on different days.

### 4.4. Gas Chromatography–Mass Spectrometry

The GC-MS analysis employed the method described by Molina-Calle et al. [65], with minor modifications. Analyses were conducted using a GC System Network Series (Agilent Technologies 7890B, Wilmington, DE, USA) coupled to a 5975C triple-axis mass selective detector, equipped with an HP-5ms 19091S-433 capillary column (J&W Scientific, Folsom, CA, USA; 30 m × 250 µm × 0.25 µm film thickness). The injector temperature was kept at 180 °C, and samples were introduced in split mode (1:5 ratio). Helium carrier gas was used at a constant flow rate of 1 mL/min (linear velocity: 27.458 cm/s). The oven temperature program began at 40 °C (held for 5 min), followed by a 10 °C/min ramp to 250 °C (held for 5 min). The total analysis time was 31 min, with a 3 min solvent delay to re-equilibrate the system conditions. Manual injections of 2 μL were performed for all samples. Electron ionization was conducted at 70 eV, with mass spectra obtained over an *m*/*z* range of 30–500. Chromatographic data were processed using MS ChemStation E.02.02.1431 and MS Interpreter 2.0 software (Agilent Technologies 7890B, Wilmington, DE, USA).

### 4.5. RBD-Compound Molecular Docking

Molecular docking studies were performed to characterize interactions at the receptor-binding domain (RBD) complex of the SARS-CoV-2 spike (S1) protein with compounds found via Elisa + GC-MS. Compound molecular structures were retrieved from the PubChem database https://pubchem.ncbi.nlm.nih.gov (accessed on 14 September 2024) using their respective Compound ID (CID) numbers. These structures were imported into UCSF Chimera to adjust protonation states and assign partial charges at neutral pH. Energy minimization was conducted to refine the compound conformation; then, torsional degrees of freedom were defined using AutoDock Tools. The three-dimensional structure of the spike (S) protein was obtained from the CHARMM-GUI database (ID: 6VXX-1-1-1). The protein was prepared by removing non-essential water molecules, ions, and ligands, followed by the addition of hydrogens and Gasteiger charges using AutoDock Tools.

All RBD–ACE2 interface-focused dockings were performed using AutoDock Vina v1.2.7 with the following settings: grid center (X, Y, Z) = (−47.398, −20.053, 17.466); grid size (Å) = 24 × 44 × 32; grid spacing = 0.375 Å; energy_range = 3; exhaustiveness = 24; num_modes = 1. AutoDock Vina v1.2.7 offers a well-established balance of sampling efficiency and scoring performance for medium-throughput virtual screening. Our group has previously implemented and documented Vina-based pipelines in related contexts [66]. While alternative engines (DockThor, GOLD, AutoDock4, etc.) are certainly applicable, in the present study we kept a single internally consistent pipeline (GC–MS → Vina → ADME/Tox) to ensure strict comparability across all phytochemicals. The receptor was treated as rigid and ligands as flexible; identical preparation and parameters were applied to every compound. This protocol followed the method outlined in Li and Shah [67]. To test that the search depth was sufficient, and the poses were stable, we executed independent dockings at exhaustiveness = 12, 24, and 48, repeating each condition 30 times and recording the best-ranked affinity (kcal/mol) of a redocking control system (PDB 5ACM), obtaining for exhaustiveness = 12: min −5.559; max −5.360; mean −5.498; sample SD 0.046, exhaustiveness = 24: min −5.540; max −5.360; mean −5.479; sample SD 0.052, exhaustiveness = 48: min −5.559; max −5.360; mean −5.498; sample SD 0.046. The invariance of the top pose and the near-identical score distributions between 24 and 48 support practical convergence; we, therefore, fixed exhaustiveness = 24 for all production dockings.

**Figure 4:**
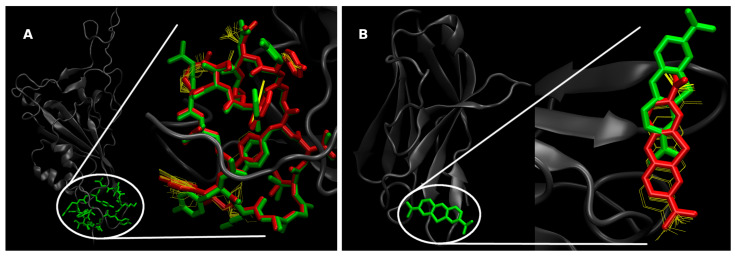
Docking protocol validation. Blind redocking of (**A**) the crystallographic ligand in PDB 7L4Z (RBD–cyclic peptide), and (**B**) methylene blue into its crystallographic target PDB 5ACM. In both panels, protein is shown in dark-grey cartoon representation; left: full protein–ligand complex; right: zoomed binding region. Crystallographic pose in green, best Vina pose in red, and additional poses from independent blind docking runs shown as yellow line traces.

For docking protocol validation (Figure 4), we first evaluated the pose recovery on the spike RBD scaffold by blind redocking the crystallographic ligand in the PDB 7L4Z (RBD–cyclic peptide, the only reported cocrystallized RBD-ligand found in the PDB at the time of this work). Using AutoDock Vina v1.2.7 (30 independent runs; grid center (X, Y, Z) = (18.766, −69.763, −52.858); grid size (Å) = 126 × 126 × 126; grid spacing = 0.375 Å; exhaustiveness = 24; num_modes = 1), the best-ranked pose reproduced the experimental geometry with RMSD = 2.01 Å (RMSD_lig-only = 9.86 Å). Affinity scores were consistent across runs (mean −8.846 kcal·mol^−1^; SD 0.117; min −9.133; max −8.571). Furthermore, we blind redocked methylene blue into its crystallographic target PDB 5ACM under the same protocol (30 independent runs; grid center (X, Y, Z) = (−2.853, 1.670, −9.274); grid size (Å) = 98 × 74 × 64; spacing 0.375 Å; exhaustiveness 24; num_modes 1), obtaining RMSD = 1.18 Å (RMSD_lig-only = 7.45 Å) and a tight affinity distribution (mean −5.980 kcal·mol^−1^; SD 0.044; min −6.040; max −5.884). These controls indicate that our preparation and search settings recover crystallographic poses and yield stable scores across independent runs.

Because the published set of small-molecule RBD–ACE2 interface blockers is currently limited, we treat the methylene blue redocking as a qualitative check rather than a quantitative correlation between ΔG and potency. We note that scores were employed only as an internal relative ranking of the garlic-derived phytochemicals under the same docking protocol. We do not interpret the score as an absolute predictor of potency. Rather, after validating the pose recovery and qualitative agreement with the known interface inhibitor, we used the scores to prioritize candidates for further study. We did not compute a decoy-set ROC/AUC due to the lack of a sufficiently powered active panel for the specific RBD–ACE2 interface.

### 4.6. Physicochemical, Pharmacokinetic, and Bioavailability Analyses

The SwissADME web tool developed by the Molecular Modeling Group of the Swiss Institute of Bioinformatics (http://www.swissadme.ch) (accessed on 14 September 2025) was used for the computational prediction of pharmacokinetic and physicochemical properties to evaluate the docking-predicted drug candidates. The platform integrates advanced predictive models to assess absorption, distribution, metabolism, and excretion (ADME) parameters alongside key molecular descriptors such as lipophilicity (iLOGP), topological polar surface area, and solubility, while employing validated frameworks like Lipinski’s Rule of Five and the BOILED-Egg model to evaluate the drug-likeness and bioavailability. Particularly valuable in natural product research, SwissADME facilitates the rapid screening of complex phytochemical libraries by finding bioactive constituents with favorable ADME profiles and filtering out compounds prone to poor absorption or metabolic instability, thereby guiding the prioritization of candidates for isolation and experimental validation. By offering early-stage insights into synthetic accessibility, blood–brain barrier penetration, and cytochrome P450 interactions, the tool streamlines lead optimization, while reducing reliance on resource-intensive in vitro assays.

## 5. Conclusions

In summary, in this study we showed that the freeze-dried Tigre cultivar garlic-derived aqueous fraction could inhibit the interaction between the SARS-CoV-2 spike receptor-binding domain (RBD) and human ACE2, suggested by a biochemical binding ELISA immunoassay. GC–MS analysis of this bioactive fraction allowed us to annotate its phytochemical composition. Docking and druggability analysis indicate that specific candidate small molecules (L17, L20, L36) are predicted to occupy a hotspot-enriched depression at the ACE2-contacting surface of the RBD, a plausible mechanism for disrupting viral entry at the host-cell attachment step.

Among these, L17 combines favorable predicted binding, interface localization, and in silico ADME/Tox properties, making it a high-priority phytochemical for experimental follow-up. Importantly, we recognize that the present study relies on in vitro binding inhibition and computational prioritization; confirmation of direct antiviral activity in a cellular pseudovirus entry model, as well as biophysical validation of RBD engagement (e.g., SPR, MST), will be essential. Future work will therefore focus on purifying or synthesizing L17, confirming its structure (NMR/MS), and testing its ability to block spike-mediated entry in relevant cellular systems.

Overall, our results support the concept that *A. sativum* contains small molecules with potential to interfere with SARS-CoV-2 spike–ACE2 recognition, and they provide chemically defined starting points for developing entry inhibitors from a widely available natural source.

## Figures and Tables

**Table 1:** *A. sativum* organosulfur compounds previously reported as having anti SARS-CoV-2 activity.

Compound	Structure	Anti-SARS-CoV-2 Target Protein	Reference
Tetrasulfide, di-2-propenyl.	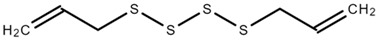	ACE2	[28]
Diallyl disulfide.	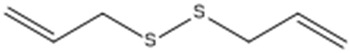	ACE2	[28]
3-Vinyl-1,2-dithiacyclohex-4-ene.	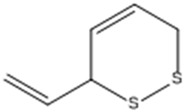	ACE2	[28]
Diallyl trisulfide.	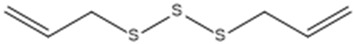	ACE2	[28]
Diallyl tetrasulfide.	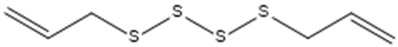	ACE2	[28]
Allicin. (Allyl 2-propenethiosulfinate).	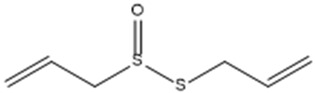	Mpro	[29]
γ-L-Glutamyl-S-allylcysteine.	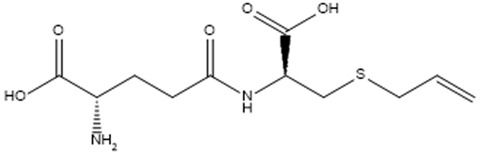	Mpro	[30]

**Table 2:** Maximum inhibition and IC_35_ ± standard error (SE), values of garlic extracts on RBD–antibody interaction. Data are reported as mean % inhibition of SARS-CoV-2 RBD binding to antibody, measured in an ELISA-format competitive binding assay. Each value represents mean of *n* = 10 independent measurements performed at the indicated extract concentration (µg/mL). Higher % inhibition indicates reduced RBD–antibody complex formation.

Extracts	Concentration (μg/mL)	Max. Inhibition (%)	IC_35_ (μg/mL)
FL	10	38.71	1.0 ± 0.38
FE	10	35.13	3.1 ± 0.62
TL	10	42.16	0.1 ± 0.03
TE	10	34.95	0.5 ± 0.06

Fermín freeze-dried (FL), Fermín oven-dried (FE), Tigre freeze-dried (TL), and Tigre oven-dried (TE).

**Table 3:** Maximum inhibition of TL-derived fractions and IC_40_ ± standard error (SE) values of fractions on RBD–antibody interaction. Data are reported as mean % inhibition of SARS-CoV-2 RBD binding to antibody, measured in an ELISA-format competitive binding assay. Each value represents mean of *n* = 10 independent measurements performed at the indicated fraction concentration (µg/mL). Higher % inhibition indicates reduced RBD–antibody complex formation.

Fractions	Concentration (μg/mL)	Inhibition Max (%)	IC_40_ (μg/mL)
Hexane	100	48.76	0.09 ± 0.03
Chloroform	100	40.36	96.07 ± 35.36
AcOEt	100	40.78	0.07 ± 0.03
Aqueous	100	57.26	0.01 ± 0.005

**Table 4:** Compounds found by GC-MS analysis of the TL bulbs’ aqueous fraction.

L	2D Structures	IUPAC	Class	Molecular Formula	Exact Mass	PubChem CID	Rt (min)	*m*/*z* Experimental [M-H]-	*m*/*z* Calculated [M-H]-	Area (%)	Reference
1	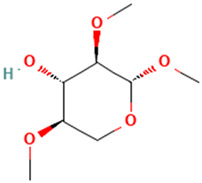	Methyl 2,4-di-O-methyl-β-d-xylopyranoside	Sugars	C_8_H_16_O_5_	192.09977361 Da	21607730	3.591	192	192.099	1.189	PubChem CID: 21607730 NIST Mass Spectrometry Data Center
2	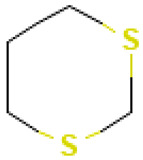	1,3-Dithiane	Sulfur compounds	C_4_H_8_S_2_	120.00674260 Da	10451	5.615	120	120	0.519	PubChem CID: 10451 NIST Mass Spectrometry Data Center
3	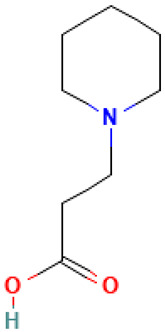	1-Piperidinepropanoic acid	Organic acids	C_8_H_15_NO_2_	157.110278721 Da	117782	6.565	157	157.11	0.107	PubChem CID: 117782 NIST Mass Spectrometry Data Center
4	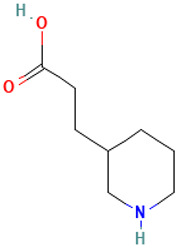	3-(Piperidin-3-yl)propanoic acid	Organic acids	C_8_H_15_NO_2_	157.110278721 Da	5152304	6.6	157	157.11	0.124	PubChem CID: 5152304 NIST Mass Spectrometry Data Center
5	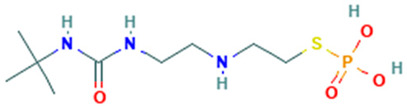	N-t-Butyl-N′-2-[2-thiophosphatoethyl]aminoethylurea	Sulfur compound	C_9_H_22_N_3_O_4_PS	299.10686436 Da	546889	6.897	166	166.06	0.166	PubChem CID: 546889 NIST Mass Spectrometry Data Center
6	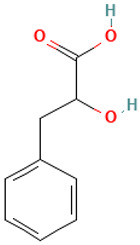	DL-3-Phenyllactic acid	Organic acids	C_9_H_10_O_3_	166.062994177 Da	3848	7.134	166	166.06	0.395	PubChem CID: 117782 NIST Mass Spectrometry Data Center
7	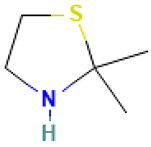	2,2-Dimethylthiazolidine	Sulfur compound	C_5_H_11_NS	117.06122053 Da	88015	7.235	117	117.06	0.476	PubChem CID: 3848 NIST Mass Spectrometry Data Center
8	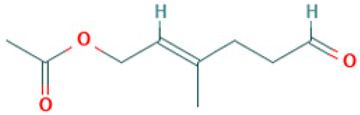	Acetic acid, 3-methyl-6-oxo-hex-2-enyl ester	Esters	C_9_H_14_O_3_	170.094294304 Da	5363568	7.657	170	170.09	1.177	PubChem CID: 5363568 NIST Mass Spectrometry Data Center
9	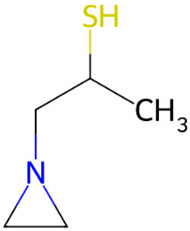	1-[N-Aziridyl]propane-2-thiol	Sulfur compounds	C_5_H_11_NS	117.06122053 Da	284764	7.959	117	117.06	0.852	PubChem CID: 284764 NIST Mass Spectrometry Data Center
10	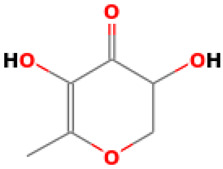	4H-Pyran-4-one, 2,3-dihydro-3,5-dihydroxy-6-methyl	Others	C_6_H_8_O_4_	144.04225873 Da	119838	8.227	144	144.04	0.467	PubChem CID: 119838 NIST Mass Spectrometry Data Center
11	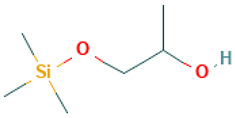	1-[(Trimethylsilyl)oxy]propan-2-ol	Others	C_6_H_16_O_2_Si	148.091956283 Da	23105108	8.494	148	148.09	4.749	PubChem CID: 23105108 NIST Mass Spectrometry Data Center
12	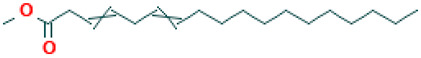	3,6-Octadecadiynoic acid, methyl ester	Esters	C_19_H_30_O_2_	294.255880323 Da	71438608	10.167	290	290.22	1.365	PubChem CID: 71438608 NIST Mass Spectrometry Data Center
13	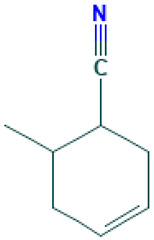	3-Cyclohexen-1-nitrile, 6-methyl	Others	C_8_H_11_N	121.089149355 Da	549257	10.547	121	121.08	0.409	PubChem CID: 549257 NIST Mass Spectrometry Data Center
14	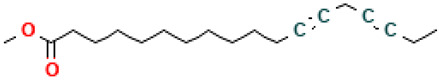	12,15-Octadecadiynoic acid, methyl ester	Esters	C_19_H_30_O_2_	290.224580195 Da	538453	10.66	290	290.22	0.409	PubChem CID: 538453 NIST Mass Spectrometry Data Center
15	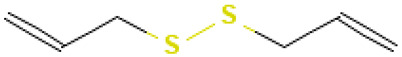	Diallyl disulphide	Sulfur compounds	C_6_H_10_S_2_	146.02239267 Da	16590	11.918	146	146.02	0.589	PubChem CID: 16590 NIST Mass Spectrometry Data Center
16	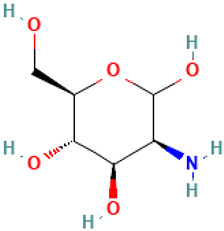	Mannosamine	Others	C_6_H_13_NO_5_	179.07937252 Da	440049	12.233	179	179.07	0.428	PubChem CID: 440049 NIST Mass Spectrometry Data Center
17	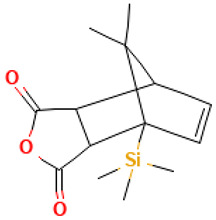	4-Oxatricyclo[5.2.1.0(2,6)]dec-8-ene-3,5-dione, 10,10-dimethyl-7-(trimethylsilyl)	Others	C_14_H_20_O_3_Si	264.11817103 Da	553289	14.732	264	264.11	2.308	PubChem CID: 553289 NIST Mass Spectrometry Data Center
18	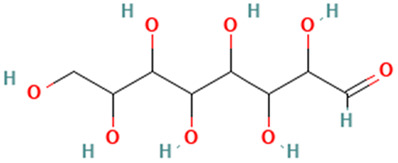	l-Gala-l-ido-octose	Sugars	C_8_H_16_O_8_	240.084517 Da	219659	12.678	240	240.08	0.651	PubChem CID: 219659; NIST Mass Spectrometry Data Center
19	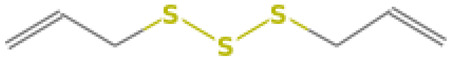	Trisulfide, di-2-propenyl	Sulfur compounds	C_6_H_10_S_3_	177.99446384 Da	16315	13.052	178	177.99	0.247	PubChem CID: 16315; NIST Mass Spectrometry Data Center
20	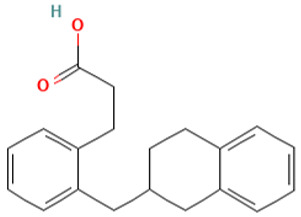	Hydrocinnamic acid, o-[(1,2,3,4-tetrahydro-2-naphthyl)methyl]	Organic acids	C_21_H_24_O_2_	294.161979940 Da	582809	15.948	294	294.16	1.503	PubChem CID: 582934 NIST Mass Spectrometry Data Center
21	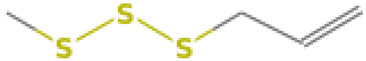	Trisulfide, methyl 2-propenyl	Sulfur compounds	C_4_H_8_S_3_	151.97881378 Da	61926	13.936	152	151.97	3.861	PubChem CID: 135403803 NIST Mass Spectrometry Data Center
22	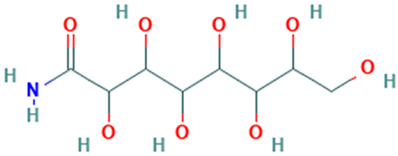	d-Gala-l-ido-octonic amide	Others	C_8_H_17_NO_8_	255.09541650 Da	552061	20.133	255	255.09	0.521	PubChem CID: 552061NIST Mass Spectrometry Data Center
23	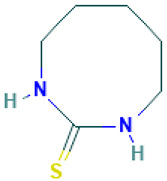	1,3-Diazacyclooctane-2-thione	Sulfur compounds	C_6_H_12_N_2_S	144.07211956 Da	5372734	14.465	144	144.07	2.114	PubChem CID: 5372734 NIST Mass Spectrometry Data Center
24	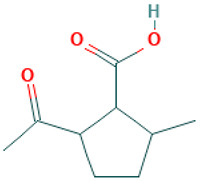	Cyclopentanecarboxylic acid, 2-acetyl-5-methyl-	Organic acids	C_9_H_14_O_3_	170.094294304 Da	538095	12.447	170	170.09	0.572	PubChem CID: 440049; NIST Mass Spectrometry Data Center
25	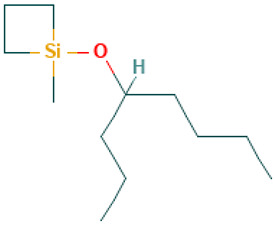	4-Methyl(trimethylene)silyloxyoctane	Others	C_12_H_26_OSi	214.175291983 Da	588574	15.153	214	214.17	2.522	PubChem CID: 588574 NIST Mass Spectrometry Data Center
26	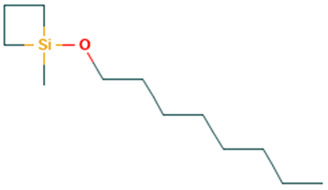	1-Methyl-1-n-octyloxy-1-silacyclobutane	Others	C_12_H_26_OSi	214.175291983 Da	598585	15.414	214	214.17	2.076	PubChem CID: 598585NIST Mass Spectrometry Data Center
27	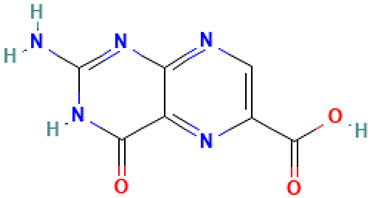	Pterin-6-carboxylic acid	Organic acids	C_7_H_5_N_5_O_3_	207.03923904 Da	135403803	13.408	207	207.03	0.182	PubChem CID: 61926 NIST Mass Spectrometry Data Center
28	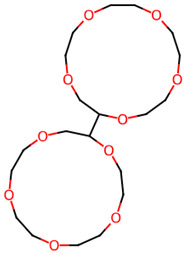	(2S,2′S)-2,2′-Bis[1,4,7,10,13-pentaoxacyclopentadecane]	Others	C_20_H_38_O_10_	438.24649740 Da	552595	16.026	438	438.24	1.712	PubChem CID: 552595 NIST Mass Spectrometry Data Center
29	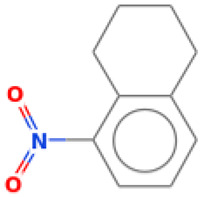	Naphthalene, 1,2,3,4-tetrahydro-5-nitro	Others	C_10_H_11_NO_2_	177.078978594 Da	93130	16.405	177	177.07	2.55	PubChem CID: 93130 NIST Mass Spectrometry Data Center
30	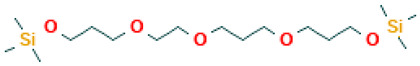	3,7,11,14,18-Pentaoxa-2,19-disilaeicosane, 2,2,19,19-tetramethyl-	Others	C_17_H_40_O_5_Si_2_	380.24142744 Da	552943	16.904	380	380.24	3.723	PubChem CID: 552943 NIST Mass Spectrometry Data Center
31	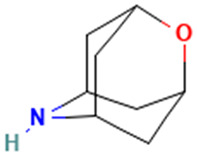	2-Oxa-6-azatricyclo [3.3.1.1(3,7)]decane	Others	C_8_H_13_NO	139.099714038 Da	586977	17.587	139	139.09	1.602	PubChem CID: 586977 NIST Mass Spectrometry Data Center
32	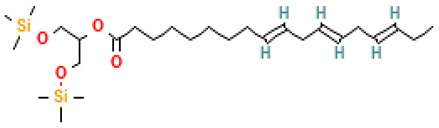	9,12,15-Octadecatrienoic acid, 2-[(trimethylsilyl)oxy]-1-[[(trimethylsilyl)oxy]methyl]ethyl ester, (Z,Z,Z)-	Esters	C_27_H_52_O_4_Si_2_	496.34041320 Da	5362857	17.925	496	496.3	0.621	PubChem CID: 5362857 NIST Mass Spectrometry Data Center
33	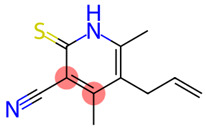	Pyridine-3-carbonitrile, 5-allyl-4,6-dimeethyl-2-mercapto-	Sulfur compounds	C_11_H_12_N_2_S	204.07211956 Da	657927	18.079	204	204.07	0.622	PubChem CID: 657927 NIST Mass Spectrometry Data Center
34	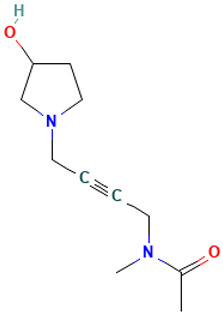	Acetamide, N-methyl-N-[4-(3-hydroxypyrrolidinyl)-2-butynyl]-	Others	C_11_H_18_N_2_O_2_	210.136827821 Da	536669	18.88	210	210.13	0.604	PubChem CID: 536669 NIST Mass Spectrometry Data Center
35	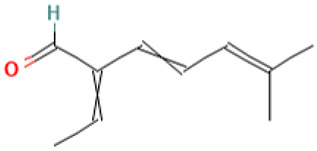	3,5-Heptadienal, 2-ethylidene-6-methyl-	Others	C_10_H_14_O	150.104465066 Da	572127	19.717	150	150.1	1.569	PubChem CID: 572127 NIST Mass Spectrometry Data Center
36	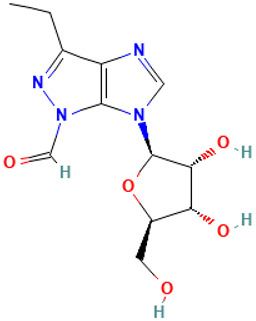	Pyrazole[4,5-b]imidazole, 1-formyl-3-ethyl-6-β-d-ribofuranosyl-	Others	C_12_H_16_N_4_O_5_	296.11206962 Da	91692119	14.612	296	296.11	1.547	PubChem CID: 91692119 NIST Mass Spectrometry Data Center
37	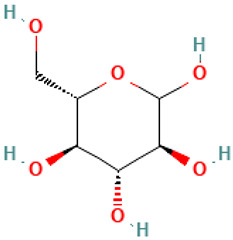	L-Glucose	Sugars	C_6_H_12_O_6_	180.06338810 Da	2724488	20.441	180	180.06	0.512	PubChem CID: 2724488 NIST Mass Spectrometry Data Center
38	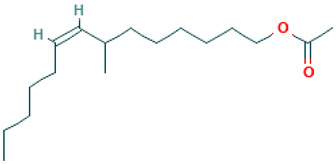	7-Methyl-Z-tetradecen-1-ol acetate	Esters	C_17_H_32_O_2_	268.240230259 Da	5363222	20.744	268	268.24	0.451	PubChem CID: 5363222 NIST Mass Spectrometry Data Center
39	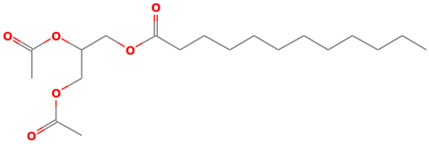	Dodecanoic acid, 2,3-bis(acetyloxy)propyl ester	Esters	C_19_H_34_O_6_	358.23553880 Da	169212	21.462	358	358.23	2.143	PubChem CID: 169212 NIST Mass Spectrometry Data Center
40	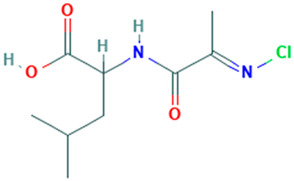	DL-Leucine, N-[2-(chloroimino)-1-oxopropyl]	Amino acid	C_9_H_15_ClN_2_O_3_	234.0771200 Da	9603629	21.694	234	234.07	1.39	PubChem CID: 9603629 NIST Mass Spectrometry Data Center
41	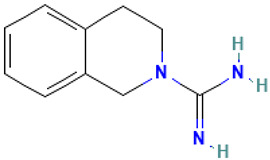	2(1H)-Isoquinolinecarboximidamide, 3,4-dihydro-	Others	C_10_H_13_N_3_	175.110947427 Da	2966	21.996	175	175.11	2.246	PubChem CID: 2966 NIST Mass Spectrometry Data Center
42	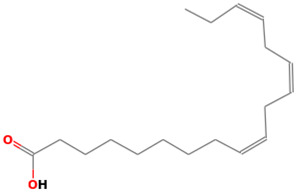	9,12,15-Octadecatrienoic acid	Organic acids	C_18_H_30_O_2_	278.224580195 Da	88801875	22.186	278	278.42	2.472	PubChem CID: 88801875 NIST Mass Spectrometry Data Center
43	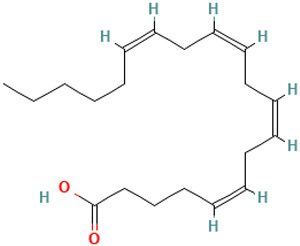	Arachidonic acid	Organic acids	C_20_H_32_O_2_	304.240230259 Da	444899	22.685	304	304.46	1.984	PubChem CID: 444899 NIST Mass Spectrometry Data Center
44	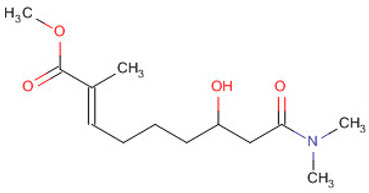	2-Nonenoic acid, 9-(dimethylamino)-7-hydroxy-2-methyl-9-oxo-, methyl ester	Esters	C_13_H_23_NO_4_	257.16270821 Da	5364160	23	257	257.16	1.579	PubChem CID: 5364160 NIST Mass Spectrometry Data Center
45	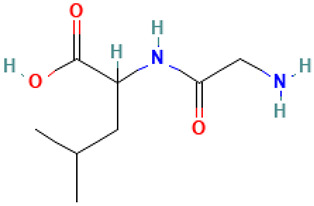	DL-Leucine, N-glycyl	Amino acid	C_8_H_16_N_2_O_3_	188.11609238 Da	102468	23.409	188	188.11	4.755	PubChem CID: 102468 NIST Mass Spectrometry Data Center
46	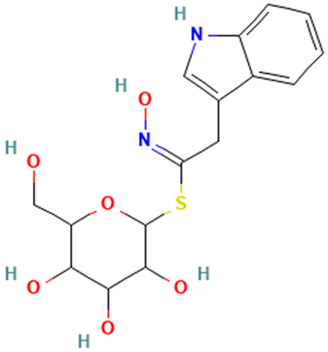	1-S-[(1E)-N-Hydroxy-2-(1H-indol-3-yl)ethanimidoyl]-1-thiohexopyranose	Sulfur compounds	C_16_H_20_N_2_O_6_S	368.10420754 Da	9603283	23.86	368	368.1	1.128	PubChem CID: 9603283 NIST Mass Spectrometry Data Center
47	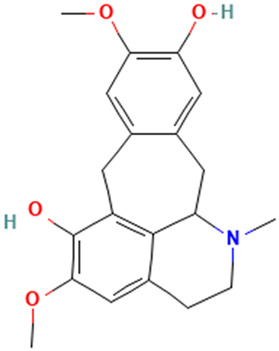	Benzocycloheptano[2,3,4-I,j]isoquinoline, 4,5,6,6a-tetrahydro-1,9-dihydroxy-2,10-dimethoxy-5-methyl	Alkaloid	C_20_H_23_NO_4_	341.16270821 Da	339326	24.014	341	341.16	0.964	PubChem CID: 339326 NIST Mass Spectrometry Data Center
48	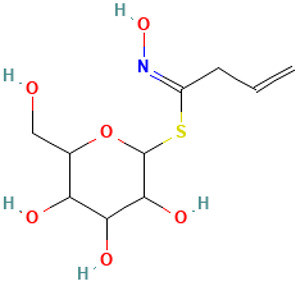	Desulphosinigrin	Sulfur compounds	C_10_H_17_NO_6_S	279.07765844 Da	9601716	25.041	279	279.07	1.055	PubChem CID: 9601716 NIST Mass Spectrometry Data Center
49	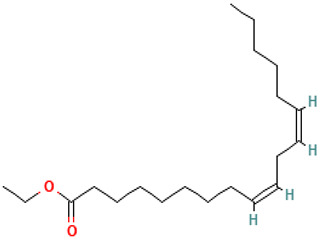	Linoleic acid ethyl ester	Esters	C_20_H_36_O_2_	308.271530387 Da	5282184	25.386	308	308.27	3.485	PubChem CID: 5282184 NIST Mass Spectrometry Data Center
50	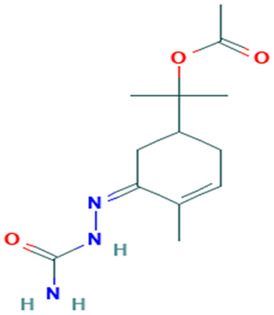	(+-)-5-(1-Acetoxy-1-methylethyl)-2-methyl-2-cyclohexen-1-one semicarbazone	Others	C_13_H_21_N_3_O_3_	267.15829154 Da	9603716	25.932	267	267.15	3.594	PubChem CID: 9603716 NIST Mass Spectrometry Data Center
51	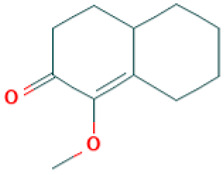	2(3H)-Naphthalenone, 4,4a,5,6,7,8-hexahydro-1-methoxy	Others	C_11_H_16_O	180.115029749 Da	534313	27.059	180	180.11	3.659	PubChem CID: 534313 NIST Mass Spectrometry Data Center
52	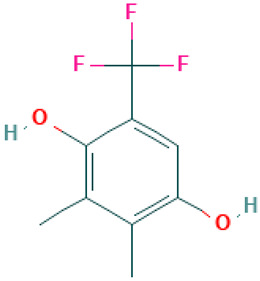	Phen-1,4-diol, 2,3-dimethyl-5-trifluoromethyl	Others	C_9_H_9_F_3_O_2_	206.05546401 Da	590850	27.558	206	206.05	2.824	PubChem CID: 590850 NIST Mass Spectrometry Data Center
53	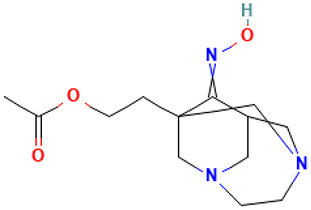	1-(2-Acetoxyethyl)-3,6-diazahomoadamantan-9-one oxime	Others	C_13_H_21_N_3_O_3_	267.15829154 Da	551906	28.561	267	267.15	4.072	PubChem CID: 551906 NIST Mass Spectrometry Data Center
54	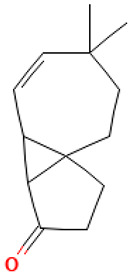	Cyclopenta[1,3]cyclopropa[1,2]cyclohepten-3(3aH)-one, 1,2,3b,6,7,8-hexahydro-6,6-dimethyl	Others	C_13_H_18_O	190.135765193 Da	561869	30.946	190	190.13	2.99	PubChem CID: 561869 NIST Mass Spectrometry Data Center
55	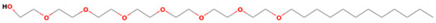	Heptaethylene glycol monododecyl ether	Others	C_26_H_54_O_8_	494.38186868 Da	76459	31.653	494	494.38	3.487	PubChem CID: 76459 NIST Mass Spectrometry Data Center

**Table 5:** Reported biological bioactivities of compounds in the *A. sativum* TL-derived aqueous fraction.

Compound	Antiviral Activity	Other Relevant Activities
L41: 2(1H)-Isoquinolinecarboximidamide, 3,4-dihydro- (Debrisoquine)	TMPRSS2 inhibitor [35]	Antihypertensive [36]
L42: 9,12,15-Octadecatrienoic acid (α-Linolenic acid)	Interrupts the binding, adsorption, and entry stages of Zika virus replication cycle [37]	Anti-obesity, antidiabetic, cardiovascular-protective, anti-inflammatory, anticancer, neuroprotection, and antibacterial [38,39] Anti-hypercholesterolemic [40]
L3: 1-Piperidinepropanoic acid	Not reported	Anti-inflammatory [41,42]
L6: DL-3-Phenyllactic acid	Not reported	Antibacterial, antibiofilm, and antifungal [43,44]
L10: 4H-Pyran-4-one, 2,3-dihydro-3,5-dihydroxy-6-methyl-	Not reported	Antioxidant [45,46,47]
L15: Diallyl disulphide (DADS)	Not reported	Anticancer [48] Cardioprotective, antihypertensive, antibacterial, antiparasitic, antioxidant, and anti-inflammatory [49]
L16: Mannosamine	Not reported	Dextransucrase inhibition [50] Cytotoxic effects with free fatty acids [51]
L19: Trisulfide, di-2-propenyl (Diallyl trisulfide or DATS)	Not reported	Antioxidant, anti-inflammatory, antibacterial, antitumor, cardioprotective, and immunomodulatory [48,52,53,54,55,56]
L21: Trisulfide, methyl 2-propenyl (MATS)	Not reported	Antiplatelet [57,58]
L43: Arachidonic acid	Not reported	Tumoricidal [59]
L47: Benzocycloheptano[2,3,4-I,j]isoquinoline, 4,5,6,6a-tetrahydro-1,9-dihydroxy-2,10-dimethoxy-5-methyl	Not reported	Antidiabetic potential [60]
L49: Linoleic acid ethyl ester (Ethyl Linoleate)	Not reported	Anti-inflammatory [61] Melanogenesis inhibitor [62,63]

**Table 6:** ADME profile of L36, L20, L17 compounds.

Compounds	L36	L20	L17
MW	368.4	294.39	264.39
H-bond acceptors	7	2	3
H-bond donors	6	1	0
iLOGP	1.33	2.73	2.49
XLOGP3	0.5	4.73	3.15
Consensus Log P	0.18	4.14	2.49
Ali Log S	−3.51	−5.24	−3.73
Ali class	Soluble	Moderately soluble	Soluble
GI absorption	Low	High	High
BBB permeant	No	Yes	Yes
Pgp substrate	No	No	No
CYP1A2 inhibitor	Yes	No	No
CYP2C19 inhibitor	No	No	No
CYP2C9 inhibitor	No	Yes	No
CYP2D6 inhibitor	No	Yes	No
CYP3A4 inhibitor	No	No	No
log Kp (cm/s)	−8.19	−4.74	−5.68
Lipinski violations	1	1	0
Ghose violations	0	0	0
Veber violations	1	0	0
Egan violations	1	0	0
Muegge violations	2	0	0
Bioavailability score	0.55	0.85	0.55
PAINS	0	0	0
Brenk	4	0	4
Leadlikeness violations	1	1	0
Synthetic accessibility	4.88	2.77	4.91

**Table 7:** Toxicity profile of L36, L20, L17 compounds.

Target	L36	L20	L17
Hepatotoxicity	Inactive (0.55)	Inactive (0.51)	Inactive (0.72)
Neurotoxicity	Inactive (0.72)	Inactive (0.61)	Inactive (0.70)
Nephrotoxicity	Active (0.57)	Active (0.54)	Inactive (0.59)
Respiratory toxicity	Active (0.74)	Active (0.62)	Active (0.67)
Cardiotoxicity	Inactive (0.60)	Inactive (0.57)	Inactive (0.83)
Carcinogenicity	Inactive (0.51)	Inactive (0.68)	Inactive (0.60)
Immunotoxicity	Inactive (0.98)	Inactive (0.99)	Inactive (0.98)
Mutagenicity	Active (0.50)	Inactive (0.66)	Inactive (0.64)
Cytotoxicity	Inactive (0.69)	Inactive (0.83)	Inactive (0.79)
BBB barrier	Active (0.59)	Active (0.81)	Active (0.89)
Ecotoxicity	Inactive (0.59)	Active (0.57)	Active (0.59)
Clinical toxicity	Inactive (0.52)	Inactive (0.55)	Inactive (0.56)
Nutritional toxicity	Inactive (0.58)	Inactive (0.65)	Inactive (0.54)

## Data Availability

The original contributions presented in this study are included in the article. Further inquiries can be directed to the corresponding author(s).
